# Effects and limitations of an automated external defibrillator with audiovisual feedback for cardiopulmonary resuscitation: a randomized manikin study

**DOI:** 10.1186/cc9716

**Published:** 2011-03-11

**Authors:** H Fischer, J Gruber, S Neuhold, S Frantal, E Hochbrugger, B Steinlechner, R Greif

**Affiliations:** 1 Medical University Vienna, Austria; 2 University Hospital Bern and University of Bern, Switzerland

## Introduction

Correctly performed basic life support (BLS) and early defibrillation are the most effective measures to treat sudden cardiac arrest. Audiovisual feedback improves BLS. Automated external defibrillators (AEDs) with feedback technology may play an important role in improving CPR quality. The aim of this simulation study was to investigate whether an AED with audiovisual feedback improves CPR parameters during standard BLS performed by trained laypersons.

## Methods

With ethics committee approval and informed consent, 68 teams (two flight attendants each) performed 12 minutes of standard CPR with the AED's audiovisual feedback mechanism enabled or disabled. We recorded CPR quality parameters during resuscitation on a manikin in this open, prospective, randomized controlled trial. Between the feedback and control group we measured differences in compression depth and rate as the main outcome parameters and effective compressions, correct hand position, and incomplete decompression as secondary outcome parameters. An effective compression was defined as a compression with correct depth, hand position, and decompression.

## Results

The feedback group delivered compression rates closest to the recommended guidelines (101 ± 9 vs. 109 ± 15/minute, *P *= 0.009), more effective compressions (20 ± 18 vs. 5 ± 6%, *P *< 0.001), more compressions with correct hand position (96 ± 13 vs. 88 ± 16%, *P *< 0.001), and less leaning (21 ± 31 vs. 77 ± 33%, *P *< 0.001). However, only the control group adhered to the recommended compression depth (44 ± 7 mm vs. 39 ± 6, *P *= 0.003).

## Conclusions

Use of an AED's audiovisual feedback system improved some CPR quality parameters, thus confirming findings of earlier studies, with the notable exception of decreased compression depth, which is a key parameter that might be linked to reduced cardiac output.

